# SpliceJumper: a classification-based approach for calling splicing junctions from RNA-seq data

**DOI:** 10.1186/1471-2105-16-S17-S10

**Published:** 2015-12-07

**Authors:** Chong Chu, Xin Li, Yufeng Wu

**Affiliations:** 1 Department of Computer Science and Engineering, University of Connecticut, 371 Fairfield Way, Unit 2155, 06269 Storrs, CT, U.S.A

**Keywords:** splicing junction calling, RNA-seq, sequence reads alignment, support vector machine

## Abstract

**Background:**

Next-generation RNA sequencing technologies have been widely applied in transcriptome profiling. This facilitates further studies of gene structure and expression on the genome wide scale. It is an important step to align reads to the reference genome and call out splicing junctions for the following analysis, such as the analysis of alternative splicing and isoform construction. However, because of the existence of introns, when RNA-seq reads are aligned to the reference genome, reads can not be fully mapped at splicing sites. Thus, it is challenging to align reads and call out splicing junctions accurately.

**Results:**

In this paper, we present a classification based approach for calling splicing junctions from RNA-seq data, which is implemented in the program SpliceJumper. SpliceJumper uses a machine learning approach which combines multiple features extracted from RNA-seq data. We compare SpliceJumper with two existing RNA-seq analysis approaches, TopHat2 and MapSplice2, on both simulated and real data. Our results show that SpliceJumper outperforms TopHat2 and MapSplice2 in accuracy. The program SpliceJumper can be downloaded at https://github.com/Reedwarbler/SpliceJumper.

## Introduction

With the development of high-throughput sequencing technologies, RNA-seq has been widely applied in transcriptome profiling. This facilitates the further studies of gene structure and expression on the genome wide scale. One of the opportunities provided by RNA-seq is detecting splicing junctions. In eukaryotic genomes, splicing is a process that exons join together and introns are excluded to form the mature mRNA. Recent studies show that variations in splicing patterns are associated with Alzheimer [1] and other complex diseases [2]. Thus, detecting splicing junctions not only helps to profile transcriptomes, but also contributes to the understanding of the mechanism of some complex diseases. In this paper, we focus on calling splicing junctions from RNA-seq data of organisms that have released reference genomes.

Over the past several years, many sophisticated computational approaches for calling splicing junctions from RNA-seq data have been developed [3-6]. But it is still challenging to call out splicing junctions accurately. One difficulty is that because of the discrete nature of RNA-seq data, reads spanning splice sites cannot be fully mapped to the reference genome. Situation becomes worse when reads span three or more exons. A common strategy is to map reads that span two or more exons as split-mapped reads. Split-mapped read means that one wants to map segments of the read onto multiple disjoint genomic regions (which correspond to the exons). Most existing RNA-seq reads alignment tools, such as TopHat [3,7], MapSplice [4], STAR [8], PALMapper [9], GSNAP [10], PASS [11], and GEM [12], are able to handle split-mapped reads with large or small gaps. But because of the repeats on genome, reads errors, and the short length of unmapped segments, it is still difficult to align the unmapped segments correctly. Another challenge is that the coverage of reads is uneven, and the expression level of many transcripts is low. Thus many exons are covered by only a few or even no reads. This can make it difficult to call out junctions especially for tools relying on coverage to form exon islands. Another issue is that many tools only use part of the information contained in the reads. For example, TopHat [7] only uses reads coverage to call splicing junctions. In principle, using more information, e.g. number of discordant encompassing pairs and number of split-mapped reads, contained in the reads may make junction calling more accurate. Moreover. the past study of RNA in organisms such as humans has accumulated substantial knowledge about RNA structure. Ideally, calling splice junctions can be assisted by these prior knowledge. Thus, it is useful to develop new tools that use more information contained in RNA-seq data and also the prior knowledge about the RNA structure to achieve higher sensitivity and specificity of the called splicing junctions.

In this paper, we introduce a new approach, SpliceJumper, which uses a machine learning approach that combines multiple features extracted from RNA-seq paired-end reads. There are three steps for SplicJumper. First, we align the raw reads to genome sequence using BWA [13] and call out all the candidate splicing sites. Then, we classify the true and false splicing sites using a machine learning approach by combining several features extracted from the reads. Finally, we call out the splicing junctions with the true splicing sites, and paired-end reads are used to filter out the false ones. The main idea behind our approach is that we treat the problem of calling splicing sites as a classification problem. Then, we use the called out splicing sites to guide the re-alignments of reads that are initially not fully mapped. This allows accurate calling of splicing junctions.

There are three main aspects for SpliceJumper:

1) SpliceJumper uses a machine learning approach to call out true splicing sites by combining more features. Also, information contained in paired-end reads is used by SpliceJumper. As in many situations, it is not easy to call out splicing junctions using only one or two features. So combining more features helps to improve sensitivity and specificity. Moreover, we use an efficient classification approach to combine all these features.

2) Similar to many existing approaches, we also re-align the initially unmapped reads. The difference is that we try to re-align the unmapped parts in focal regions with the help of the called out splicing sites. This way, it not only works efficiently, but also helps to filter out ambiguous alignments. We show that SpliceJumper outperforms TopHat2 and MapSplice2 in accuracy on both simulation and real data.

3) Unlike tools such as TopHat, MapSplice, or other tools that require user-provided threshold parameters to call out splicing junctions, SpliceJumper learns the parameters through the training procedure. Thus there is no need for users to set any thresholds.

## Background

### Signatures of splicing junctions on reads

**Figure 1:**
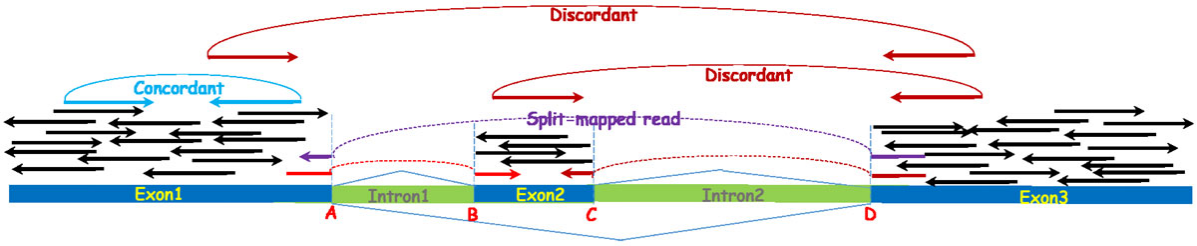
**Features indicating existence of splicing junctions: discordant encompassing pairs, split-mapped reads, and coverage change at two neighbor regions of a splicing site**. A, B, C, and D are four splicing sites.

Alternative splicing is a regulated process during gene expression that results in a single gene coding for multiple proteins. In this process, some exons of a gene may be included within, or excluded from, the final processed messenger RNA (mRNA) produced from that gene [14]. Each alternative splicing event happens at a donor site and an acceptor site. And these two splicing sites form a splicing junction. When sequenced reads are taken from a splicing site, various signatures left by the splicing junction may be found from the reads. Figure 1 illustrates an alternative splicing event. A, B, C, and D are four splicing sites, and three splicing junctions, [A,B], [C,D], and [A,D], are formed.

There are mainly three types of signatures that can be extracted from the RNA-seq reads around splicing sites.

(i) Discordant encompassing pair signature: the number of discordant encompassing pairs. A discordant pair has insert size from the two mapped reads outside the range [m-3v,m+3v], where m is the mean insert size, and v is the standard variation of the insert size. If the length of a splicing junction is large enough, then a pair-end reads encompassing the splicing junction will become discordant after aligned to the reference genome. Figure 1 shows two discordant pairs that encompass splicing junctions [C,D] and [A,D].

(ii) Split-mapped reads signature: the number of split-mapped reads. Reads spanning splicing sites may be clip-mapped at the splicing sites. Thus, each read will be split into two or more segments. If all these segments are aligned correctly, the split-mapped read may reveal the positions of the splicing sites. Figure 1 shows three split-mapped reads that span splicing sites A, B, C, and D. Take the read split-mapped at sites A and B as an example. The partially mapped segment (the left segment) clipped at site A indicates site A is a potential splicing site. And the clipped segment (the right segment) can be mapped at site B that indicates site B is also a potential splicing site.

(iii) Coverage changing signature: the coverage change between left and right neighboring regions. Coverage change will be apparent from left neighboring region to right neighbor region of a splicing site. This is because no reads will be mapped at the right region of a donnor site, and no reads aligned at the left region of an acceptor site. Figure 1 shows the coverage changing of the four splicing sites A, B, C, and D.

More detailed information of parsing features from RNA-seq data are explained in the Method section.

### Existing approaches

During the past few years, many tools have been developed to call splicing junctions. According to features used and strategies to combine features, there are two main types of approaches. The first is "exon inference" based approach, which uses coverage information to infer exons first, and then align initially unmapped reads and call splicing junctions. One tool is TopHat which first infers exon islands with the initially mapped reads aligned by Bowtie [15,16]. Then TopHat concatenates the potential exons using the known splicing motifs, and finally re-aligns the initially unmapped reads to the jointed exons. Another similar tool is PASSION [6]. PASSION also builds exon islands from initially mapped reads. Then it uses the pattern growth algorithm to split and align unmapped reads. And finally a filtering strategy is used to call out splicing junctions. When coverage is high, this kind of approaches is expected to work well. However, when coverage is low, these approaches may not work well. For example, because of the existence of errors (reads errors or alignment errors), variations, or the uneven coverage, there may be no reads covering some (small or large) regions of an exon. Thus the exon will be partitioned into two or even more "exons" in this step. This not only affects the alignments of reads in the following steps, but also introduces many false positives.

The second type of approach is "gap alignment" based. There are two main steps for this kind of approaches. The first step is a "seed-extend" process that splits reads into segments (called kmers), which are then aligned to the reference genome independently. If a segment cannot be initially mapped, sometimes its neighboring segments are mapped. If this segment can be reconstructed by extending its neighboring regions, the gap spanned by this segment is considered as a potential splicing junction and is called out. In the second step, true splicing junctions are called out according to some threshold parameters. MapSplice is a tool of this kind. In the second step, each potential splicing junction is scored by MapSplice according to anchor significance and entropy. Another similar approach is TopHat2, which is an improved version of TopHat. One difference between TopHat2 and MapSplice is that TopHat2 first aligns reads to transcriptome that are generated from provided annotations. It is believed that "gap alignment" based approaches perform better than "exon inference" based methods, especially when the expression level or coverage is low [17]. However, this kind of approaches also has its own limitations. For example, the length of many splicing junctions is several thousands bases or even larger, and many kmers may be ambiguously mapped in such a long region. Thus it can be difficult for those tools to distinguish which is the true alignment, even using some anchor based strategies.

Besides these two main kinds of approaches, there are other approaches that combine different features, such as TrueSight [5]. TrueSight combines RNA-seq read mapping quality and coding potential of genomic sequence into a model, which is trained by iterative logistic regression. Then TrueSight uses the model to de novo identify splicing junctions and filter out unreliable ones. One issue is that TrueSight views each candidate junction as a whole and this may introduce false positives. For example, if the donor site is of high confidence (have strong features) while the acceptor site is a false one, then it is quite possible for TrueSight to call this splicing junction as a true one. This can introduce false positives.

In this paper, we compare the performance of SpliceJumper with two tools. One is TopHat2. TopHat2 is widely used for splicing junction calling and RNA-seq reads alignment. The other is MapSplice. MapSplice performs well for both reads alignments and splicing junction calling according to two assessment papers [17,18].

## Methods

### High-level approach

Our approach, SpliceJumper, aims to call splicing junctions from RNA-seq paired-end reads, and also align the reads. There are mainly three steps to call out the splicing junctions. First, SpliceJumper aligns the raw reads to the reference sequence using BWA, and calls out all the candidate splicing sites. As each candidate splicing site is either a true one or a false one, this problem can be viewed as a classification problem. So it is natural to use machine learning approaches to perform classification. Thus in the second step we use a supervised machine learning approach to call splicing sites. We choose supervised machine learning because for both real and simulation data, we have enough labeled data as training data. To train a model, choosing the appropriate features is important. SpliceJumper uses four features parsed from the three signatures explained in the Background Section. After parsing all the features, SpliceJumper trains a model using the training data. Then with the trained model, SpliceJumper calls out the splicing sites. Finally, in the third step, we call out the splicing junctions with the called out splicing sites. In this step, each called out splicing junction should satisfy three conditions: 1)Both the donor and acceptor sites are the true ones that are called out in the second step. 2) At least one read is split-mapped at the donor and acceptor sides. 3) The distance between the mapped segment and the clipped segment is concordant with the insert size between the split-mapped read and its mapped mate read. And also, the reads alignment is finished during this procedure.

### Details of our approach

SpliceJumper calls splicing junctions in three steps. In the first step, BWA is used to align the raw reads to the reference genome and from the BAM file we call out all the candidate splicing sites. Then we collect all the features of each candidate splicing site, train a SVM model based on training data, and then call out the true ones using the trained model. Finally, we call out splicing junctions with the true splicing sites.

#### Preprocessing and candidate splicing sites calling

SpliceJumper requires BAM files (sorted and indexed) as input. So before calling candidate splicing sites, BWA (or other alignment tools reporting soft-clipped and hard-clipped alignments) is used to align the raw reads to the reference genome. Then reads are classified to three types according to the alignment type: fully mapped reads, clip-mapped reads including soft-clipped and hard-clipped reads, and other reads. Fully mapped reads refer to the reads that can be fully aligned to the reference. BWA only reports the primary alignment. So if only a part of a read is primarily aligned, the read will be reported as a clip-mapped read. Depending on whether the clipped part is ambiguously mapped or not, clip-mapped reads are classified as hard-clipped reads and soft-clipped reads. Other reads are mainly unmapped reads, which usually refer to those reads that are mapped to the junction of two adjacent reference sequences. In our approach, reads with low mapping quality or unmapped are discarded. During the process of classifying reads, reads coverage is calculated. If a read pair is discordant, we also mark the positions of the read pair.

**Figure 2:**
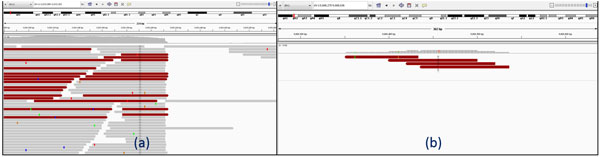
**Two types of candidate splicing sites: Figure (a) shows candidate splicing sites called out by clipped reads; Figure (b) shows candidate splicing sites called out by coverage changing**.

To call candidate splicing sites, our approach is based on two observations: 1) For splicing sites with enough reads covered, when aligned to the reference genome, reads will be clip-mapped at the splicing sites. 2) For splicing sites without enough reads covered, there may be no reads clip-mapped at the splicing sites. But the read coverage will change near the splicing sites. For example, maybe there is only one read fully mapped within an exon, and we can call out this type of candidate splicing sites through coverage change. If read coverage at some sites decreases to zero or increases from zero, then this site is also called out as a candidate junction site. Thus, two types of candidate sites are called out at this stage: one is the read clip position, and the other is the coverage change position. Figure 2 shows an example of the two types of candidate splicing sites. Figure 2(a) is a type one candidate splicing site. We can see many reads are clipped at the splicing sites. Figure 2(b) is a type two candidate splicing site, which does not have enough reads covered, and no reads clipped at the splicing sites, but there is coverage change. Candidate splicing sites within s bases (by default s = 10) from each other are combined. SpliceJumper provides a "-s" option for users to set s. Each candidate site is given a direction to indicate whether it is an candidate donor site or candidate acceptor site. For the first type of candidate sites, site direction is decided by the reads clip direction. If most of the reads are left-segment clipped, then it is a candidate donor site. Otherwise it is a candidate acceptor site. For the second type of candidate sites, if coverage changes from zero to some value then it is an acceptor site. Otherwise it is a donor site. Note that not all splicing sites will be called out at this step. Some of these missed splicing sites may still be called during the reads re-alignment step.

#### Feature parsing

Once the candidate splicing sites are called out, SpliceJumper collects features for each site. We extract four features from the three signatures mentioned in the Background Section. A clipped read contains two types of segments: partially mapped segments and clipped segments. Different types of segments provide information for different splicing sites. So we collect the two features from the split-mapped reads signature: (i) the number of reads clipped at candidate sites, and (ii) the number of clipped segments mapped at candidate sites. From the other two signatures we collect two features: (iii) the number of discordant encompassing paired-end reads, and (iv) the coverage difference between the left and right neighbor regions. Thus, each candidate site will be represented by four features and be viewed as a point in four dimensional space. In this way, the splicing sites calling problem is converted to a classification problem.

(i) Reads clipped at candidate sites: the number of reads clipped at candidate sites. Because of the existence of introns, reads spanning the splicing sites cannot be fully mapped and are reported as soft-clipped or hard-clipped alignments. This provides us not only the positions of splicing sites, but also strong evidence that the candidate splicing sites may be the true ones. Figure 1 shows three reads clipped at splicing sites.

(ii) Clipped segments mapped at candidate sites: the number of clipped segments, which initially cannot find primary alignments when aligned with BWA, but are mapped at candidate splicing sites in the re-alignment stage.

**Figure 3:**
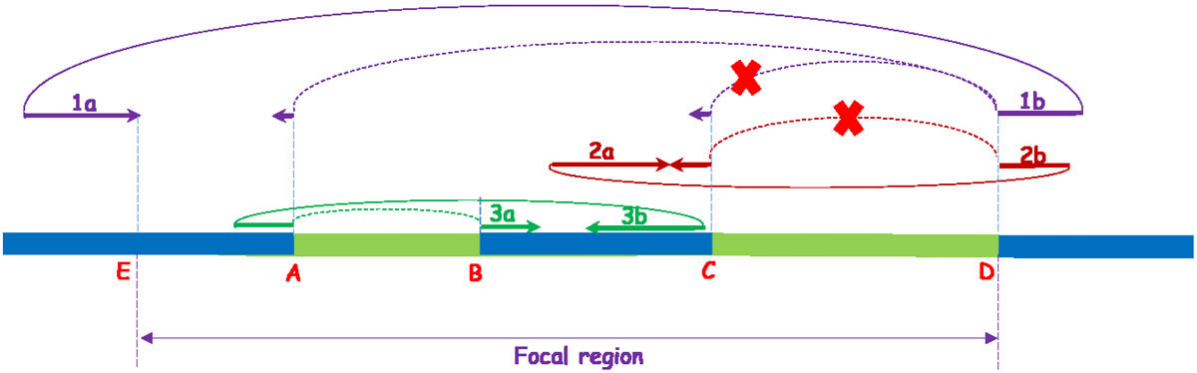
**Illustration of paired-end reads that are used to guide the alignment and filter out false splicing junctions**. A, B, C and D are four called out splicing sites. 1(1a and 1b), 2(2a and 2b), and 3(3a and 3b) are three pairs. 1a, 2a, and 3b are fully mapped, while 1b, 2b, and 3a are split-mapped. The right end of 1a is aligned at site E, and [E,D] forms a focal region to align the clipped segment of 1b. Insert size between 1a and 1b can be used to guide the alignment of the clipped segment of 1b: the clipped segment is short and can be aligned at both position A and C, but the alignment at C is wrong because otherwise 1a and 1b form a discordant pair which has low probability. Thus alignment at position A is chosen. Paired-end reads can also be used to filter out false splicing junctions. Splicing junction [C,D] is considered as a false one, because although read 2b is split-mapped at position C and D, 2a and 2b will form a discordant pair if the alignment of 2b is correct. Thus a conflict happens, and SpliceJumper will report [C,D] a false positive. In contrast, splicing junction [A,B] is considered as a true splicing junction.

To obtain the quantity of this feature, we re-align the clipped segments to the reference genome. For hard-clipped reads, because the clipped segments are not reported in the alignments, we trace the original reads from the raw read files (in fastq format) according to the "qname" field of alignments. Thus all hard-clipped reads are transformed to soft-clipped reads. Then for each soft-clipped read, we do re-alignment based on the observation that if a read is clipped at one side of a junction, then the clipped segment should align at the other side of the junction. So for the suffix soft-clipped reads, we align the clipped segment to candidate acceptor sites following down the reference genome. And for prefix soft-clipped reads, we try to align them to candidate donor sites following up the reference genome. If both the suffix and prefix of a read are clipped, then it is quite likely that the read spans more than two exons. In this case, we align both the suffix and prefix clipped segments separately following the same strategy. Suppose the read is clipped at *b*_0_, and we use [*b*_1_, *b*_2_] to indicate the region where to search the alignment. The insert size of paired-end reads is used to bound the searching. If the suffix (or prefix) segment of a read is clipped, and the mate read is mapped at the right (or left) side of this read, then the mapping position *b_p _*of the mate read can be used as the right (or the left) boundary of the searching. Then the searching region becomes [*b*_0_, *b_p_*] (or [*b_p_, b*_0_]). Figure 3 shows an example of how paired-end reads are used to guide the alignments. 1a and 1b are a pair, and 1a is fully mapped at E (right end of 1a) while 1b is clipped at position D. Thus, [E,D] forms a focal region to align the clipped segment of 1b. Because 1b is clipped at an acceptor site, we try to align the clipped segment at donor sites in region [E,D]. Because the clipped segment is short, we find two alignments at A and C. The insert size between 1a and 1b here is used to call out the correct one. The alignment at C is likey to be false because otherwise 1a and 1b will form a discordant pair. But this is not very likely and so the alignment at position A is chosen.

If no paired-end reads can be used as the boundary, a maximum junction size with default value 1,500,000 bp is used as the boundary. For reads clipped at donor (acceptor) sites, the searching region is [*b*_0_,*b*_0_+1500000] ([*b*_0_-1500000,*b*_0_]). We try to find an alignment around related splicing sites in the searching region. Here, "related" means for reads clipped at donor sites, we only check candidate acceptor sites; and conversely for reads clipped at acceptor sites, we only check candidate donor sites. If an alignment can still not be found, then we try to align the segment within the whole region [*b*_1_, *b*_2_] using local alignment. And if the segment finds an alignment at position *p_new _*, then *p_new _*will be viewed as a new candidate splicing site and is added into the candidate splicing sites list. If the clipped segment finds an alignment at candidate site *c_j _*, then the number of clipped segments mapped at *c_j _*is increased by one.

(i) Discordant encompassing pair: the number of discordant encompassing pairs.

Recall that a discordant pair has insert size from the two mapped reads outside the range [m-3v,m+3v], where m is the mean insert size, and v is the standard variation of the insert size. See Figure 1 for an illustration of discordant encompassing pair for splicing junctions. Note that not all the junctions can make encompassing read pairs to be discordant. This is because if the length of a splicing junction is smaller than 3v, then even the read pair encompasses the junction, it still could be concordant. Our approach relies on other features to call out these splicing junctions. According to our experiments, around nine tenths of the junctions can cause encompassing pairs to be discordant. Thus, discordant encompassing pair is still a useful feature for calling splicing junctions.

(ii) Coverage difference between left and right neighboring regions: for candidate donor (acceptor) sites, it is the average coverage of left (right) region minus the average coverage of right (left) region. The average coverage of a region is calculated by: $\sum_{i=1}^{L_{d}}\left(d_{i}\right)/L_{d}$, where *L_d _*is the length of the region and *d_i _*is the read depth at position *i *of the region. SpliceJumper provides a "-l" option for users to set the region length (with default value 25 bp). Figure 1 illustrates the coverage difference at splicing sites A, B, C and D.

#### Classification and call out junctions

After parsing all the features for each candidate splicing site, SpliceJumper uses a machine learning based approach to classify all the candidate splicing sites into the true ones and the false ones. Before training the model, for each candidate site with four features we perform normalization. This is based on the observation that coverage is quite uneven. And for a candidate site from a region with high coverage the quantity of the features will be large, while for a candidate site from a region with low coverage the quantity will be small. However, maybe both of the two sites are the true ones. Thus using the original quantity of the features may mislead the classifier. So normalization before training can improve the accuracy. For candidate donor sites, all the four features are normalized by the average coverage of the left neighboring regions. For candidate acceptor sites, all are normalized by the average coverage of the right neighboring regions.

*Model training and classification *We use support vector machine (SVM) to perform classification. In particular, we use LibSVM [19] to train a model, and then use the trained model to classify splicing sites. To train a model, training data that contains candidate sites and labeled with true or false should be provided. For simulation data, the true label of each candidate is known. So to prepare the training data we just label the candidate splicing sites that are ture as 1, and the rest are labeled as 0. For real data, users can train the model with released annotations, such as human annotations released by UCSC, Ensembl, Geneid, Genscan, RefSeq, SGP, AceView, Vega, etc. One problem is that not all transcripts will express in every sample. In other words although some splicing sites do exist in annotations, they may not express. So there will be no reads cover those splicing sites. This kind of splicing sites should not be considered as true ones. So before using labeled splicing sites released in annotations, first we check whether the coverage around the sites is zero or not. If larger than zero then the sites are labeled with true. In this way, we get all the positive cases of the training data. Then we randomly choose the same number of negative cases, which are sites that have reads covered but not in annotation. With the positive and negative cases, we have all the training data of real data. To train a model, 10-cross validation and grid search are used to find the optimal parameters. Then with the trained model, we classify the candidate sites in testing data into the true and false ones.

*Junction calling *After finishing classification, SpliceJumper has called out all the true splicing sites. There are two steps to call out the splicing junctions. First, we call out all the candidate splicing junctions. To call a splicing junction, first both the two sites (one is donor and the other is acceptor) of the junction should be true in classification. There should be a connection between the two sites. We say two sites are connected if there at least one read that is clipped at the donor (acceptor) site, and the clipped segment of the read can be aligned at the acceptor (donor) site. In practice, this step is finished in feature parsing step, and a graph with all the candidate sites as nodes and connecting edges is kept. In the second step, we check whether there is a conflict between the insert size and the mapping position of the clipped segment. If the mapped clipped segment leads the read pair to be discordant, then we say it is a conflict. If conflicts happen, it is quite possible that the connection between the two sites is false. This may be caused by wrong alignment of the clipped segment because the segment usually is short and there are repeats in genome. We discard this kind of splicing junctions. Figure 3 shows an example: A, B, C, and D are called out splicing sites. 1(1a and 1b), 2(2a and 2b), and 3(3a and 3b) are three pairs. 1a, 2a, and 3b are fully mapped, while 1b, 2b, and 3a are split-mapped. Splicing junction [C,D] is considered as a false one, because although read 2b is split-mapped at position C and D, 2a and 2b will form a discordant pair if the alignment of 2b is correct. Thus a conflict happens, and SpliceJumper will report [C,D] as false positive. In contrast, splicing junction [A,B] is considered as a true splicing junction.

## Results

We run SpliceJumper on both simulation and real data, and compare with TopHat 2.0.10 and MapSplice 2.1.6 on accuracy and efficiency. For simulation data, because we know the true junctions, we can calculate the number of true positive, false positive and false negative of each tool. Three metrics are used: 1) Precision=TP/(TP+FP), 2) Recall=TP/(TP+FN), and 3) F-value = 2*Precision*Recall/(Precission+Recall), where TP represents true positive, FP represents false positive, and FN represents false negative. For real data, because no true junctions are provided, we cannot directly evaluate the accuracy of called junctions of each tool. We rely on another metric: the ratio of mapped bases. RNA-seq reads alignment and splicing junction calling are related to each other. To call splicing junctions accurately the first step is to align reads correctly, and at the same time calling splicing junctions accurately can also help to guide the alignment of the unmapped reads. Thus, the ratio of mapped bases is an approximate estimate of the accuracy of called out splicing junctions. For real data, as the origin of each read is unknown, we give the ratio of mapped bases. For simulation data, because the true alignment position of each read is known, we compare the ratio of correctly mapped bases.

### Calling RNA junctions with simulated data

We use the same simulated data released in [17]. Two datasets (Test1 and Test2 of simulation one) are used. All the reads are simulated using a tool BEERS released in the same paper. Both of the datasets are generated from 30,000 mouse build mm9 transcript models. Indel, substitution and error rates for the Test1 dataset are 0.0005, 0.001 and 0.005 respectively, and 0.0025, 0.005 and 0.01 for Test2 dataset. For each dataset, 10 million pairs of reads with read length 100bp are simulated. Wetest the accuracy of the three tools on chromosome 11. It is unfair for all the tools if there are no reads covering the splicing sites. So when calculating the accuracy, only junctions with at least one read covered are counted. Thus there are 14,939 and 15,738 benchmarked junctions on chromosome 11 for Test1 and Test2 dataset respectively. Recall that SpliceJumper requires training data. We use the splicing sites of chomosome 1 as the training data, and test the performance of the trained model on chromosome 11.

To compare the called out junctions with the benchmark data, a slack value is introduced. If the distance between both the left and right sides of a called out splicing junction and a benchmarked one is smaller or equal to the slack value, then the called out one is considered as a correct one. Table 1 shows the change of F-value of the three tools as slack value increases from 0 to 15 for the Test1 and Test2 dataset. The results show that the F-value improves for all the three tools when the slack value increases. When the slack value is 0, MapSplice2 has the best performance but none of the three tools reaches its best performance. And when the slack value reaches 8, the F-value of all the three tools basically reaches stable values, and SpliceJumper has the best performance. Detailed results for the three tools when the slack value is 8 are shown in Table 2 and Table 3 for Test1 and Test2 dataset respectively.

**Table 1:** Comparison of SpliceJumper, TopHat2 and MapSplice2 on simulation data.

Slack value	Test1 dataset			Test2 dataset		
	**SpliceJumper**	**TopHat2**	**MapSplice2**	**SpliceJumper**	**TopHat2**	**MapSplice2**

0	0.8314	0.0003	0.8918	0.7940	0.0015	0.8288
1	0.8923	0.0016	0.9627	0.8548	0.0103	0.9319
2	0.9367	0.9370	0.9631	0.9156	0.8558	0.9328
3	0.9618	0.9377	0.9639	0.9400	0.9016	0.9471
4	0.9666	0.9384	0.9642	0.9491	0.9150	0.9473
5	0.9676	0.9390	0.9647	0.9495	0.9156	0.9476
6	0.9676	0.9396	0.9650	0.9562	0.9166	0.9485
7	0.9676	0.9397	0.9650	0.9573	0.9167	0.9502
8	0.9676	0.9397	0.9651	0.9578	0.9178	0.9508
9	0.9677	0.9397	0.9651	0.9578	0.9178	0.9508
10	0.9677	0.9398	0.9651	0.9578	0.9178	0.9508
11	0.9677	0.9398	0.9651	0.9578	0.9179	0.9510
12	0.9678	0.9399	0.9651	0.9578	0.9179	0.9510
13	0.9678	0.9399	0.9652	0.9579	0.9180	0.9510
14	0.9678	0.9399	0.9652	0.9579	0.9180	0.9510
15	0.9678	0.9400	0.9653	0.9579	0.9180	0.9510

Slack value is introduced when comparing a called out junction with a benchmarked one. And if the distance of both sides between the called out one and the benchmarked one is within the slack value then the called out one is considered as a correct one. We compare the F-value (2*Precision*Recall/(Precision+Recall)) of the three tools for different slack values. The higher the F-value the better the performance.

**Table 2:** Comparison of SpliceJumper, TopHat2 and MapSplice2 on simulated Test1 dataset when the slack value is 8.

Tools	False positive	False negative	True positive	Precision	Recall	F-value
SpliceJumper	78	862	14,077	0.9945	0.9423	0.9677
TopHat2	248	1,475	13,430	0.9819	0.9010	0.9397
MapSplice2	125	888	14,006	0.9912	0.9404	0.9651

False positive: the number of called out junctions that are actually false ones. False negative: the number of not called out junctions that are actually true ones. True positive: the number of called out junctions that are actually true ones.

**Table 3:** Comparison of SpliceJumper, TopHat2 and MapSplice2 on simulated Test2 dataset when the slack value is 8.

Tools	False positive	False negative	True positive	Precision	Recall	F-value
SpliceJumper	334	969	14,769	0.9779	0.9384	0.9578
TopHat2	717	1,784	13,954	0.9511	0.8866	0.9178
MapSplice2	358	1,151	14,587	0.9760	0.9269	0.9508

False positive: the number of called out junctions that are actually false ones. False negative: the number of not called out junctions that are actually true ones. True positive: the number of called out junctions that are actually true ones.

We also calculate the ratio of correctly mapped bases. For Test1 dataset, Splice-Jumper is 95.10%, while TopHat2 and MapSplice2 are 93.49% and 94.11% respectively. For Test2 dataset, SpliceJumper is 92.09%, while TopHat2 and MapSplice2 are 89.76% and 91.25% respectively.

### Calling RNA junctions with real data

The real data is released in [20], which is gathered across a time-course experiment (GEO accession number: GSM818582). There are 65,352,789 pairs of reads with read length 101 bp. We compare the performance of the three tools on chromosome 11. We train the model based on the annotation released by UCSC. 8,000 splicing sites of chromosome 20 in the released annotation data are used as positive cases, and each splicing site has at least one read covered. 8,000 randomly generated sites are used as negative cases, and each site has coverage larger than zero and is not included in released annotation. SpliceJumper calls out 9,902 junctions, while TopHat2 and MapSplice2 calls out 9,836 and 9,878 junctions respectively. Because the true junctions are unknown, we use the ratio of mapped bases to evaluate the performance of the three tools. The ratio of mapped bases of SpliceJumper is 92.69%, while 90.89% and 92.13% for TopHat2 and MapSplice2 respectively. One can see SpliceJumper has the highest ratio of mapped bases.

We also calculate the number of overlapped junctions of the two tools. Similar to the junction comparison in simulation section, a slack value is also introduced when checking whether one splicing junction of one tool overlaps with a splicing junction of another tool. Detailed results of number of overlapped splicing junctions as slack value increases are shown in Table 4. From the results, we see that when the slack value is 7, the number of called out junctions of all the three tools converges. Splice-Jumper has 8,205 junctions overlapping with MapSplice2, and has 7,726 junctions overlapping with TopHat2, while TopHat2 has 7,526 junctions overlapping with MapSplice2.

**Table 4:** Comparison of SpliceJumper, TopHat2 and MapSplice2 on real data.

Slack value	SpliceJumper with MapSplice2	SpliceJumper with TopHat2	TopHat2 with MapSplice2
0	7,346	4,534	4,601
1	7,491	5,128	4,987
2	7,769	6,254	5,589
3	8,201	6,675	6,701
4	8,204	7,302	7,465
5	8,204	7,701	7,509
6	8,205	7,726	7,526
7	8,205	7,729	7,526
8	8,205	7,729	7,526
9	8,205	7,729	7,526
10	8,205	7,729	7,526

Slack value is introduced when comparing two junctions. If the difference between both the left sides and the right sides of two splicing junctions is smaller or equal to the slack value, then we consider these two splicing junctions overlap with each other. When the slack value reaches 7, all the three tools give stable results.

### Running time and memory usage

We benchmark the running time and memory usage on simulated Test1 dataset. The server configuration is: eight core CPU (Intel(R) Xeon(R) X5482 @ 3.20 GHz) with 32G memory. SpliceJumper accepts bam files aligned by BWA (or other tools which report soft-clipped and hard-clipped alignments). If only original raw reads are provided, users can run BWA (or other tools) to do alignment first. To make the fair evaluation, besides the running time of SpliceJumper, we also include the running time of BWA, model training time and predicting time. Detailed running time and memory usage are provided in Table 5. The results show that the running time for the three tools are similar, and MapSplice2 is a little better. For memory usage, SpliceJumper is better.

**Table 5:** Comparison of running time and memory usage for SpliceJumper, TopHat2 and MapSplice2 on simulated Test1 dataset.

Tools	Running time	Memory
SpliceJumper	711 minutes and 28 seconds	3.14G
TopHat2	679 minutes and 18 seconds	6.53G
MapSplice2	548 minutes and 5 seconds	4.61G

## Discussion

**Figure 4:**
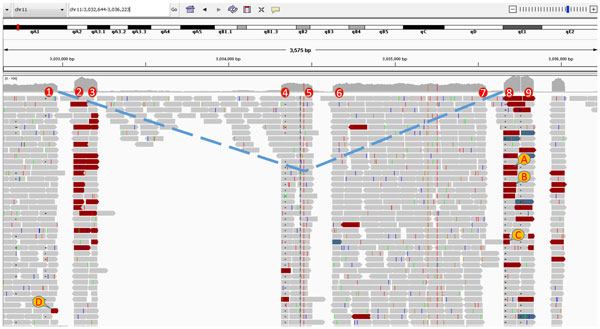
**Sequence reads from 3,032,644 to 3,036,223 on chromosome 11 of simulated Test1 dataset**. A benchmarked splicing junction from 3,033,210 to 3,035,657 (connected by dash lines) is called out by SpliceJumper, but missed by both TopHat2 and MapSplice2. 1-9 are nine splicing sites. A, B, and C are three split-mapped reads (only mapped part shown) that are clipped at splicing site 8, and the clipped segment can be aligned at site 1. D is clipped at splicing site 1. The clipped segment can be mapped at site 8. A, B, C, and D and their mate reads form four discordant pairs encompassing splicing site 1 and site 8.

We show in this paper that SpliceJumper is more accurate than TopHat2 and Map-Splice2 on both the simulated and real datasets for calling splicing junctions. Splice-Jumper achieves higher accuracy due to three improvements. First, SpliceJumper views each splicing site independently, and extracts more features for each splicing site. For example, SpliceJumper uses the discordant encompassing pair feature that is not used by TopHat2 and MapSplice2. This feature is a strong feature to indicate the existence of splicing junctions. Second, SpliceJumper uses a machine learning approach to combine the features, which not only uses the features in a more effective way, but also avoids the threshold parameters setting issue. Finally, with the called out splicing sites, the clipped segments can be aligned in focal regions, which improves the accuracy. For illustration, we show an example from the simulated Test1 dataset analyzed in the Results Section. Figure 4 shows the IGV [21] view of a region from 3,032,644 to 3,036,223 of chromosome 11. The splicing sites are labeled from 1 to 9. We can see that all the nine splicing sites have strong features: many reads are clipped at the splicing sites; there is significant coverage change between the two neighbor regions of the splicing sites; and many discordant read pairs encompass the splicing sites. SpliceJumer calls out all the marked nine splicing sites correctly. Reads marked as A, B, C, and D are clipped and also discordant with their mate reads. For example, the "cigar" field for A and B are "14S10M1I75M" and "21S10M1I68M" respectively, and the mate read mapping positions for A and B are 3,032,234 and 3,032,253 respectively. From the benchmark data, we know there is a junction from 3,033,210 to 3,035,657 which indicates an alternative splicing event at site 1 and site 8. SpliceJumper correctly calls out this splicing junction, while neither TopHat2 nor MapSplice2 calls out this junction. One reason is that for the clipped reads such as A or B, the clipped segment is short (14 bp for A and 21 bp for B), and the region between site 1 and site 8 is long (2,447 bp). Also there are three exons between them. So it is quite possible for the clipped segments aligned to ambiguous positions that neither TopHat2 nor MapSplice2 can distinguish. In contrast, SpliceJumper first tries to align the clipped segments to focal regions, not the whole region. Consider the read A in the above example. Because it is clipped at an acceptor site. So to align the clipped segment we only check the focal regions of site 7, 5, 3, and then 1. Even when the clipped segment is short, SpliceJumper is still able to align it to the correct position.

One issue of using SpliceJumper is that SpliceJumper needs training data (i.e. data with known splicing sites). For well studied organisms, we can use the released annotation data to generate training data. In the case when no annotation data is available, we believe that simulated data may also be used as training data to train models for real data analysis. In [22], we show that models on simulated data can give reasonably accurate insertion and deletion genotype calling on real data.

For future work, we notice that some kinds of structural variations, like long deletions, can introduce false positives to our results. Many of the existing methods such as TopHat2 and MapSplcie2 can call indels while aligning reads. So in the future work, we plan to add the indels calling part, which is not only useful to profile the gene structure, and also to improve the accuracy of splicing junctions calling.

## Competing interests

The authors declare that they have no competing interests.

## Authors' contributions

CC designed algorithms, developed software, performed analysis and experiments, wrote the paper. XL performed analysis and experiments. YW designed the algorithms, wrote the paper and supervised the project. All authors have read and approved the final manuscript.

## Declarations

Publication of this article was funded by National Science Foundation grant IIS-0953563.

This article has been published as part of *BMC Bioinformatics *Volume 16 Supplement 17, 2015: Selected articles from the Fourth IEEE International Conference on Computational Advances in Bio and medical Sciences (ICCABS 2014): Bioinformatics. The full contents of the supplement are available online at http://www.biomedcentral.com/bmcbioinformatics/supplements/16/S17.
